# Supplementary material for: HIVprotI: an integrated web based platform for prediction and design of HIV proteins inhibitors
Source: J Cheminform. 2018 Mar 9;10:12. doi: 10.1186/s13321-018-0266-y (PMC5845081; doi:10.1186/s13321-018-0266-y)
Supplement: Supplementary file 2 — Additional file 2. Source code of HIVProtI web server. [file 13321_2018_266_MOESM2_ESM.zip › HIVprotI_Source-code/hivprotI/weka-3-6-6/doc/weka/gui/treevisualizer/TreeBuild.html]

TreeBuild


---


|  |  |  |
| --- | --- | --- |
| **Overview** \| **Package** \| **Class** \| **Tree** \| **Deprecated** \| **Index** \| **Help** \| **Weka's home** | |  |
| **PREV CLASS**   **NEXT CLASS** | **FRAMES**    **NO FRAMES**     **All Classes** |  |
| SUMMARY: NESTED \| FIELD \| CONSTR \| METHOD | DETAIL: FIELD \| CONSTR \| METHOD |  |


---


## weka.gui.treevisualizer Class TreeBuild

```
java.lang.Object
  weka.gui.treevisualizer.TreeBuild
```

---

``` public class TreeBuild extends java.lang.Object ```

This class will parse a dotty file and construct a tree structure from it
with Edge's and Node's

**Version:**
:   $Revision: 7059 $

**Author:**
:   Malcolm Ware (mfw4@cs.waikato.ac.nz)

---

**Constructor Summary**

`TreeBuild()`
  
          Upon construction this will only setup the color table for quick
reference of a color.


| **Method Summary** | |
| --- | --- |
| `Node` | `create(java.io.Reader t)`             This will build A node structure from the dotty format passed. |

**Methods inherited from class java.lang.Object**

`equals, getClass, hashCode, notify, notifyAll, toString, wait, wait, wait`

**Constructor Detail**

### TreeBuild

```
public TreeBuild()
```

:   Upon construction this will only setup the color table for quick
    reference of a color.


**Method Detail**

### create

```
public Node create(java.io.Reader t)
```

:   This will build A node structure from the dotty format passed. Don't
    send a dotty format with multiple parents
    per node, and ensure that there is 1 and only 1 node with no parent.

    :   **Parameters:**: `t` - The reader with the dotty string to be read. **Returns:**: The top node of the tree structure (the last node with no parent).


---


|  |  |  |
| --- | --- | --- |
| **Overview** \| **Package** \| **Class** \| **Tree** \| **Deprecated** \| **Index** \| **Help** \| **Weka's home** | |  |
| **PREV CLASS**   **NEXT CLASS** | **FRAMES**    **NO FRAMES**     **All Classes** |  |
| SUMMARY: NESTED \| FIELD \| CONSTR \| METHOD | DETAIL: FIELD \| CONSTR \| METHOD |  |


---
